# Decreasing the blood flow of non-compressible intra-abdominal organs with non-invasive transcutaneous electrical stimulation

**DOI:** 10.1038/s41598-024-55165-8

**Published:** 2024-05-02

**Authors:** Yusuf O. Cakmak, Prashanna Khwaounjoo, Joseph Pangilinan, Innes K. Wise, Chris Burrows, Pranish Kolakshyapati, Zoe Williams, Paul Bannon

**Affiliations:** 1 Point-of-Care Technologies Theme, Centre for Bioengineering and Nanotechnology, Dunedin, New Zealand; 2 Interventional Technologies Theme, Medical Technologies Centre of Research Excellence, Auckland, New Zealand; 3 Centre for Health Systems and Technologies, University of Otago, Dunedin, New Zealand; 4 Brain Health Research Centre, University of Otago, Dunedin, New Zealand; 5 Cakmak Lab, Department of Anatomy, School of Biomedical Sciences, University of Otago, PO BOX 56, Dunedin, 9054 New Zealand; 6 Hybrid Theatre, Charles Perkins Centre, The University of Sydney, Sydney, Australia; 7 Cardiovascular Surgery, Sydney Medical School, Sydney, Australia; 8 The Baird Institute of Applied Heart & Lung Surgical Research, Sydney, Australia; 9 Department of Cardiothoracic Surgery, Royal Prince Alfred Hospital, Sydney, Australia; 10 Auckland Bioengineering Institute, University of Auckland, Auckland, New Zealand

**Keywords:** Neuromodulation, Blood flow, Non-invasive, Non-compressible, Blood-shift, TENS, Preclinical research, Neuro-vascular interactions

## Abstract

Non-invasive neuromodulation of non-compressible internal organs has significant potential for internal organ bleeding and blood-shift in aero/space medicine. The present study aims to investigate the potential influences of the non-invasive transcutaneous electrical nerve stimulation (TENS) on multiple non-compressible internal organs’ blood flow. Porcine animal model (n = 8) was randomized for a total of 48 neuromodulation sessions with two different TENS stimulation frequencies (80 Hz, 10 Hz) and a placebo stimulation. A combination of two different electrode configurations (Abdominal-only or Abdominal and hind limb) were also performed. Intraarterial blood flow measurements were taken during pre and post-stimulation periods at the left renal artery, common hepatic artery, and left coronary artery. Intracranial, and extracranial arterial blood flows were also assessed with digital subtraction angiography. TENS with abdominal-only electrode configurations at 10 Hz demonstrated significant reductions in average peak blood flow velocity (APV) of the common hepatic artery (p = 0.0233) and renal arteries (p = 0.0493). Arterial pressures (p = 0.0221) were also significantly lower when renal APV was reduced. The outcome of the present study emphasises the potential use of TENS in decreasing the blood flow of non-compressible internal organs when the correct combination of electrodes configuration and frequency is used.

## Introduction

Non-compressible internal organ bleeding is a life-threatening condition and current treatment approaches are invasive and require interventions. Non-invasive electrical stimulation is a promising approach to decrease non-compressible internal organ blood flow and can be activated remotely or autonomously in emergency situations allowing additional time for medical teams to arrive and provide interventions. Another potential use for non-invasive blood modulation of internal organs is the mitigation of the blood shift that threatens multiple organs in extreme conditions, especially in aviation and space medicine.

In the last two decades, numerous studies, including those from our group, have investigated the potential neuromodulator effects of non-invasive electrical skin stimulation on non-compressible internal organ flow^1–7^. These studies demonstrated that the frequency and electrode configuration (dermatome) are significant parameters of effective non-invasive transcutaneous stimulation to induce vascular responses of the targeted internal organ’s arteries^1–7^. The sympathetic nerves in the dermatomes and their spinal and supra-spinal reflex responses in the corresponding organ’s arterial smooth muscle innervation have been proposed as the main mechanisms of action^1–7^.

Steiner-Victorin et al. demonstrated that percutaneous electrical stimulation with 10 Hz and 80 Hz has opposite effects on ovarian blood flow in animals^1–3^. In the following years, our group demonstrated that lower abdominal skin (T12) stimulation with 10 Hz can increase the blood flow to testicular arteries in healthy humans^7^. We also demonstrated that the same stimulation parameters are incapable of modulating the testicular arteries’ blood flow if the electrodes are configured in a different abdominal dermatome (T10). In addition, our group also demonstrated the same approach can increase the perfusion in torsional testis (180 degrees) in animals^5^. Our further digital modelling studies on testicular torsion also demonstrated the same stimulation can be effective up to 270 degrees of testicular torsion^8^. Furthermore, it has also been shown that percutaneous electrical stimulation of L1 to S2 spinal nerve territories increased uterine artery blood flow in humans^9^. Finally, our group has also shown that 80 Hz percutaneous abdominal electrical stimulation has the potential to decrease uterine blood flow in healthy women^4^ and in myoma^6^. These studies were all performed under the pain threshold intensity and internal organs’ arterial blood flow modulations were recorded with Doppler ultrasound.

Invasive electrical stimulation studies have also used the same target (smooth muscles of the non-compressible organ’s arteries) to decrease the internal organs’ blood flow^10–12^. Invasive electrical stimulation of arteries, or in other words, directly stimulating the arteries by placing the electrodes on these arteries demonstrated that 10 Hz electrical stimulation can constrict the arteries in animal models^11^, however, the intensity of electrical stimulation in these studies was too high as 150 V which may not be applicable for non-invasive approaches. In this context, frequency-specific effects are confounded by the high intensities and the authors also demonstrated that low versus high voltage effects may have varying mechanisms of action in animals^10–12^.

Within these non-invasive and invasive approaches, researchers monitored the response of the targeted internal organ’s arteries, however there is also a possibility of blood flow modulation (vasodilation or vasoconstriction) in the neighbouring organs. Remote organs' (including cerebral and cardiac) blood flow has the potential to be modulated in these invasive and non-invasive approaches in the context of “remote conditioning”. Up until now, technical difficulties on blood-flow monitoring has also limited the monitorisation of multiple internal organs’ blood flow in the same sessions.

In this study, we aimed to investigate multiple organs' (Renal artery, Common Hepatic artery, Coronary artery and Carotid artery) blood flow responses to low (10 Hz) and high (80 Hz) frequencies in two different electrode configurations of transcutaneous electrical nerve stimulation (TENS) with high but acceptable intensity (35 mA) that can be used in humans^13^. To overcome technical difficulties to monitor multiple internal organ blood flow in the same session, we incorporated cutting-edge technologies to measure blood flow including intravascular flow probes and digital subtraction angiography (DSA) based flow measurement software. To our knowledge, this is the first study in which the abdominal and lower limb TENS is performed to modulate and simultaneously monitor multiple non-compressible internal organs’ intraarterial blood flow.

## Materials and methods

This study was ethically approved (2018/1375) by the University of Sydney Animal Ethics Committee and conducted in accordance to the University of Sydney Animal Ethics Committee guideline. The Institutional Animal ethics committee complies with the Australian NHMRC rules for animal experimentation and international regulations including the Declaration of Helsinki. The study is reported in accordance with ARRIVE guidelines.

### Animals

Eight healthy male white sus domesticus pigs were obtained from a commercial piggery and acclimatised in the facility for at least 7 days prior to any experiments. Solid pig weaning feed and water were made available ad libitum for the entire duration of the animals’ stay. Animals’ mean weights were weighing 52 ± 6 kg.

### Study design

Animals (n = 8) were randomly assigned to a protocol in the 8-day study. Animals were deemed healthy on pre-anaesthetic physical examination. Each animal was anaesthetised and catheterised for pressure and flow measurements. They were then stimulated as described in the “Stimulation” section and pressure/hemodynamic recordings were recorded (“Blood flow and pressure measurements” section) before and post-stimulation at multiple arterial locations (renal, hepatic, coronary, and carotid arteries). The animals were euthanized humanely with intravenous barbiturates at the end of each day.

### Experimental protocol

Animals were sedated with tiletamine/zolazepam 6 mg/kg (Zoletil®, Virbac, Australia) and dexmedetomidine 12 mcg/kg (Dexdomitor®, Zoetis, Australia). Anaesthesia was induced with 4–6 mg/kg of propofol (Propofol-Lipuro 1%®, B. Braun, Germany) intravenously. Anaesthesia was maintained with propofol 8 mg/kg/hr, ketamine 5 mg/kg/h (Ketamil®, Troy Laboratories, Australia) and dexmedetomidine 2–4 mcg/kg/hr administered intravenously via individual syringe drivers. This TIVA protocol was previously demonstrated to have negligible effects on vascular tone and blood pressure^14^.

Vital signs (pulse oximetry, invasive blood pressure, heart rate, electrocardiogram, end tidal carbon dioxide, and oesophageal temperature) were continuously monitored throughout the procedure (IntelliVue Mx800 system, Philips Healthcare, Australia). Arterial blood gas analysis was performed intermittently throughout anaesthesia to assess physiological status and ensure normal organ perfusion and ventilation.

### Stimulation

Non-invasive transcutaneous electrostimulation electrodes (TENS) were used to facilitate the electrical stimulation. A TENS stimulator (SBEX, Texas Southwest Research Institute, Texas, US) delivered stimulation in electric pulses (biphasic square waveform) with a current of 35 mA and a pulse width of 180 µs. 35 mA intensity is an acceptable high intensity for humans’^13^ and was utilised in the present protocol to be able to overcome the pig skin depth and allow the stimulation parameters to be translational for potential future clinical use. Stimulations were administered for 10 min each at three different frequencies: high frequency of 80 Hz, a low frequency of 10 Hz, and a placebo stimulation. “High” and “Low” frequency terminology is based on the previous literature for blood flow modulation (1–3,5,7) and other peripheral nerve stimulation studies that described low (< 10 Hz) to high (> 50 Hz) frequencies^15^. At least 20-min was allowed in between each stimulation to allow the return to baseline blood flow levels (where the APV was not fluctuating more than ± 3 cm/s for 2 min). The stimulation modalities/parameters were single-blinded for the research team (the imaging team were blinded for the stimulation combinations modalities/parameters).

**Figure 1:**
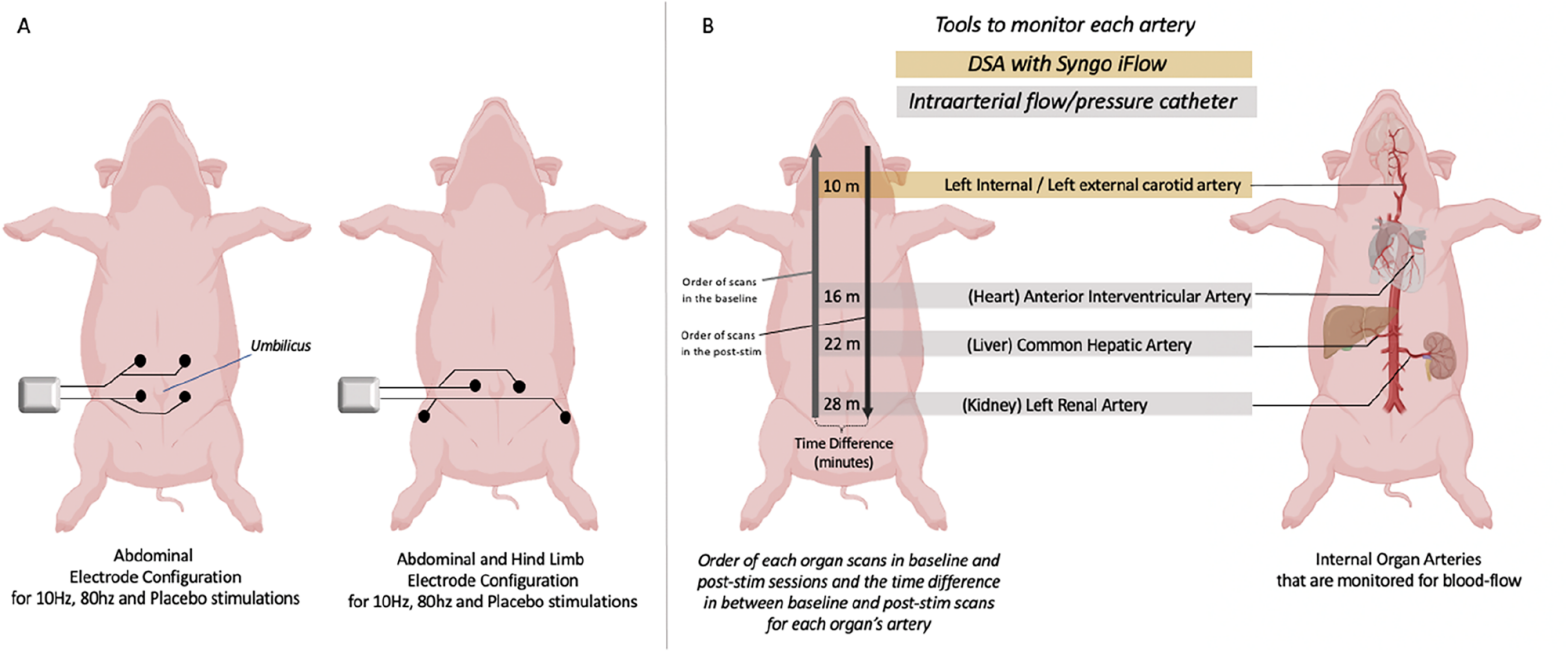
Stimulation electrode orientation and order of recordings. (**A**) Electrode orientation Abdominal (left) and Abdominal and hind limb (right). (**B**) Order and locations of pressure and flow measurements for the carotid, interventricular, hepatic and renal arteries. (Adapted from Animal figures by BioRender.com, 2021, licence University of Otago).

Three different type of stimulation parameters (80 Hz, 10 Hz and placebo) were combined with two different types of electrode configurations. These two different electrode configurations are designed as abdominal only and abdominal and hind limb (Leg) stimulation orientations (Fig. 1A).

#### Abdominal only configuration of the electrodes

For the abdominal only (Fig. 1A, left) configuration, 4 TENS electrodes were placed 2 cm inferiorly and superior to the umbilicus and 3 cm apart laterally from the midline passing through the umbilicus on each side to form a rectangle shape configuration.

This configuration is designed to match with hepatic and renal sympathetic innervation which innervates arterial smooth muscles of these organs. It has been demonstrated that the sympathetic splanchnic nerves that innervate the liver originate from neurons in the celiac and superior mesenteric ganglia, which are innervated by pre-ganglionic neurons located in the intermediolateral column of the spinal cord between T7 and T12 levels^16,17^*.* Furthermore, in pigs, there are more than 12 thoracic vertebrae (the most common is T14, in some cases T16) and their corresponding intercostal nerves. The dye injections that are performed 1 cm cranial to the pig umbilicus demonstrated the staining of T9 to T14 (intercostal) spinal nerves where all the ventral branches of spinal nerves T8 to L3 were dissected and neither the T8 nor the lumbar nerves were stained^18^*.* It is also demonstrated that lumbar branches can only be stained when the abdominal dye injections are performed caudal to the last thoracic rib in pigs^19^*.* Therefore, in the context of the umbilicus localisation at the T10 dermatome in humans and considering the umbilicus as a reliable comparative anatomical structure and the segmental abdominal innervation literature of the pigs listed above, the abdomen-only configuration of the electrodes that are placed 2 cm above and 2 cm below the umbilicus of the pigs in the present study should approximately comprise T7(T8) and T14(16) but not the lumbar segments. Hence, the abdominal-only configuration of electrodes in the present study is considered to be addressing the hepatic segmental sympathetic innervation in pigs.

In addition to the liver, the abdominal-only configuration also addresses the renal sympathetic innervation. The renal sympathetic postganglionic neurons are typically located in the dorsal root ganglia, predominantly T12-L1 in different species however, it is worth noting that renal sympathetic preganglionic neurons are located predominantly in the intermediolateral column of the spinal cord between T5 and T13 which is evidenced using retrograde tracing techniques^20^. This suggests that the abdomen-only configured electrodes segmentally correspond with renal sympathetic innervation as well.

#### Abdominal and hind limb combined configuration of the electrodes

For the abdominal and hind limb (Fig. 1A, Right) stimulation, two electrodes were placed 2 cm inferior to the umbilicus and 3 cm lateral to midline (same as the lower electrodes of the abdominal only orientation) while the other 2 electrodes were placed on the hind limbs (3 cm lateral and inferior to the knee, on the L6 dermatome).

This configuration utilised the same lower electrodes of the abdomen only configuration [2 cm below the umbilicus that corresponds to the lowest thoracic segments T14(16)] and additional electrodes that are configured on the lateral aspect of the knee which corresponds with L6 in pigs^21^. This second configuration (abdomen and hind limb) does not comprise (or very minor contribution) the nerve segments (T7(T8) to T14(16) that correspond with renal or hepatic sympathetic innervation but T14(16) and L6 segments.

Each electrode configuration and stimulation frequency were randomised across the animals to reduce confounding effects. The randomisation of the stimulations over each pig are presented in Table 1.

**Table 1:** Randomisation of stimulation protocols.

Pig	Protocol 1	Protocol 2	Protocol 1			Protocol 2		
1	AL	A	Placebo	10 Hz	80 Hz	80 Hz	Placebo	10 Hz
2	AL	A	80 Hz	10 Hz	Placebo	Placebo	10 Hz	80 Hz
3	A	AL	Placebo	80 Hz	10 Hz	80 Hz	10 Hz	Placebo
4	AL	A	80 Hz	Placebo	10 Hz	10 Hz	80 Hz	Placebo
5	A	AL	10 Hz	Placebo	80 Hz	10 Hz	Placebo	80 Hz
6	A	AL	10 Hz	80 Hz	Placebo	80 Hz	10 Hz	Placebo
7	AL	A	80 Hz	Placebo	10 Hz	Placebo	80 Hz	10 Hz
8	A	AL	10 Hz	80 Hz	Placebo	Placebo	10 Hz	80 Hz

A pig for each day (in total 8 pigs) is used for the study. (Configuration of electrodes, *AL* Abdomen and Hind Limb, *A* Abdomen only).

### Blood flow and pressure measurements

To measure the pressure and haemodynamic responses due to stimulation, the animals were catheterised with flow and pressure sensors. Two 6F sheaths were percutaneously placed in each femoral artery. One additional 7F sheath was placed in the left femoral artery for mean arterial blood pressure measurement (Arterial pressure- PA). Four 6F Cordis Hockey Sticks guide catheters were inserted through a 6F sheath for each of the following arteries (Fig. 1B), left renal, common hepatic, anterior interventricular artery, and left carotid arteries. A mixture of equal parts saline and iodine-based non-ionic contrast agent, iohexol, 647 mg/ml, equivalent to 300 mg iodine per ml (Omnipaque-300, GE Healthcare, Chicago, IL) was used via hand injection to visualize the targeted arteries for any vessel spasm and dissection.

**Figure 2:**
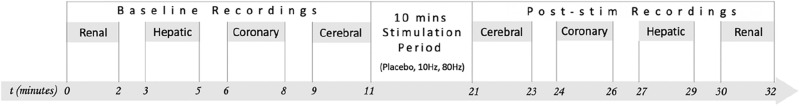
Experimental protocol and recording order for multiple organs.

Blood flow and pressure measurements were taken before and after the stimulation periods (Fig. 2). Vessels of multiple organs were chosen to investigate the effects of stimulation, these include: the left renal artery to assess renal blood flow, common hepatic artery for hepatic flow via a branch of coeliac trunk, and the left coronary artery for coronary blood flow. Care was taken to keep the wire tip at the centre of the artery lumen for optimal measurements. The positioning of the catheters for each artery was kept consistent in between sessions with the aid of the previous session’s X-ray capture.

The organ's arterial pressure (Distal pressure - PD) and flow velocity (Average peak flow velocity -APV) signals, were measured simultaneously using the Combowire® (Volcano Corporation, San Diego, CA, USA) and data recording software (Labchart, ADInstruments, Bella Vista, NSW Australia) via data acquisition hardware device (Powerlab 16/35 box, ADInstruments, Bella Vista, NSW, Australia). Simultaneous recordings of PA (via femoral artery) and ECG signals were also acquired (Phillips IntelliVue MX800, Cambridge MA, USA).

### Cerebral measurements

For cerebral (intracranial) and extracranial arterial blood flow, digital subtraction angiography (DSA) protocols were applied. The common carotid artery was injected via the catheter with 10 ml of the iodine-based non-ionic contrast agent, iohexol, 647 mg/ml, equivalent to 300 mg iodine per ml (Omnipaque-300, GE Healthcare, Chicago, IL) using a power-contrast injector (Angiomat Illumina, Liebel-Flarsheim, USA) over 2 s (flow rate 5 ml/s). The tip of the catheter was kept in the common carotid artery before and after the stimulation. The positioning of the catheters for each artery was kept consistent in between sessions with the aid of the previous session’s X-ray capture. Angiographic images were acquired at a rate of 7.5 fps in anterior–posterior, lateral position, with a robotic c-arm system (Artis Pheno, Siemens Healthineers, Erlangen, Germany). Post-processing analyses of cerebral blood flow was conducted with Syngo iFlow (Siemens Healthineers, Erlangen, Germany).

The order of flow (and pressure) recordings of each organ for each session were as follows. The baseline recordings followed the order of renal, common hepatic, coronary arteries and cerebral circulation (carotid) and post-stimulation recordings were obtained in the order of cerebral, coronary, common hepatic and renal arteries (Fig. 1B Left, Fig. 2).

In between each organ recording, there was 1 min of catheter travel time and 2 min of recording time for each organ’s artery (Fig. 2).

### Data analysis

Data sets were stored digitally and analysed offline using MATLAB (R2019b, Mathworks, MA, USA) with a custom-made software package.

#### Analysis of pressure-flow velocity-derived indices in intraarterial catheter recordings

APV in cm/s, PD in mmHg were investigated to determine stimulation-based modulation of PD and APV at renal, hepatic and coronary arteries as well as PA for the corresponding periods. Data for each experimental condition was averaged over 15 s (30 s into the session) during the recordings for each organ location. All analyses were performed in a fully automated fashion, removing the need for manual selection of data time points.

#### Intracranial and extracranial blood flow analysis with DSA-Syngo iflow

**Figure 3:**
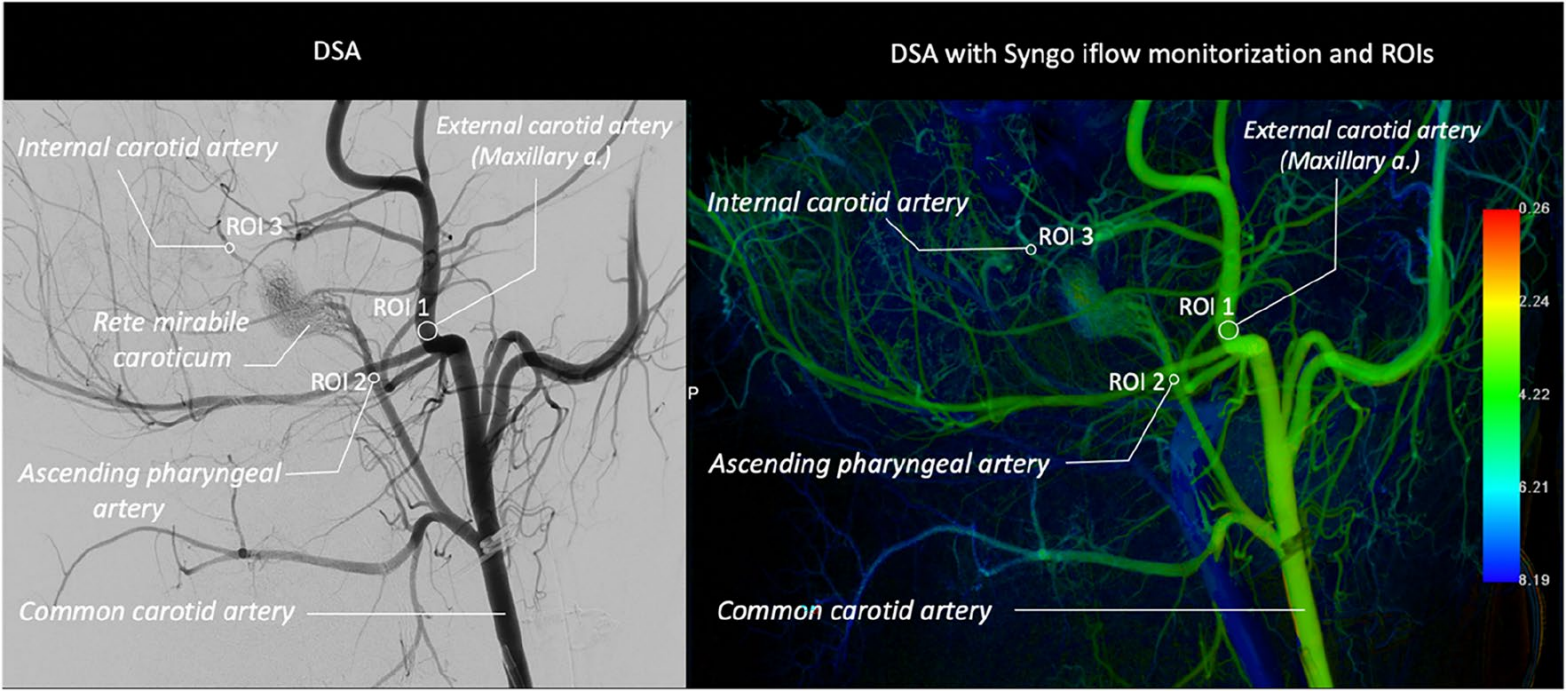
Porcine Intracranial and extracranial arterial with ROIs in DSA and DSA with iflow blood flow analyses. Colour bar represents ROI contrast peak time(s) scale.

Regions of interests (ROIs) were selected distally to the common carotid artery (CCA), ROI1 on maxillary artery, a branch of external carotid artery for the extracranial artery and the ascending pharyngeal artery (ROI2) and internal carotid artery (ICA) (ROI3) was selected for intracranial arterial blood flow analyses in Syngo iflow-color-coded DSA (Fig. 3). Each of the ROIs peak which provide indication blood flow metrics were determined using the commercially available post-processing software Syngo iFlow (Siemens Healthineers, Erlangen, Germany). For each ROI, a time-versus-intensity graph was exported by the software with calculated metrics of: (1) *ROI peak time* time for the contrast intensity of a ROI to reach peak value; (2) *ROI arrival time* the time of arrival of contrast material; (3) *MTT* mean transit time, i.e. average contrast transit time through the ROI, measured as the time difference between each of the arterial ROI peaks; and (4) *TTP* time to peak i.e. time from the first appearance of contrast in the ROI to the peak contrast concentration in the ROI.

### Statistical analyses

Shapiro–Wilk test was used to test if the data was normally distributed. All data was normally distributed. Statistical analyses were then performed in two-way ANOVA to investigate the effects of placebo, 10 Hz and 80 Hz stimulations on the characteristics of blood flow, i.e., velocity, distal pressure, and arterial pressure, quantified by the mean, standard error of the mean (SEM) and p-value. Fisher's least significant difference (LSD) test was then used to quantify the differences and computes a p-value for each comparison without adjusting for multiple comparisons. A p-value of less than 0.05 was considered to be statistically significant. GraphPad Prism Version 9.3.0 was used for all statistical analysis.

## Results

There were frequency, location and electrode orientation dependant changes in measures obtained from the pressure and flow velocity-derived indices. Changes in APV were observed for the common hepatic artery and renal artery (and also PA for renal artery).

### Common hepatic artery

**Figure 4:**
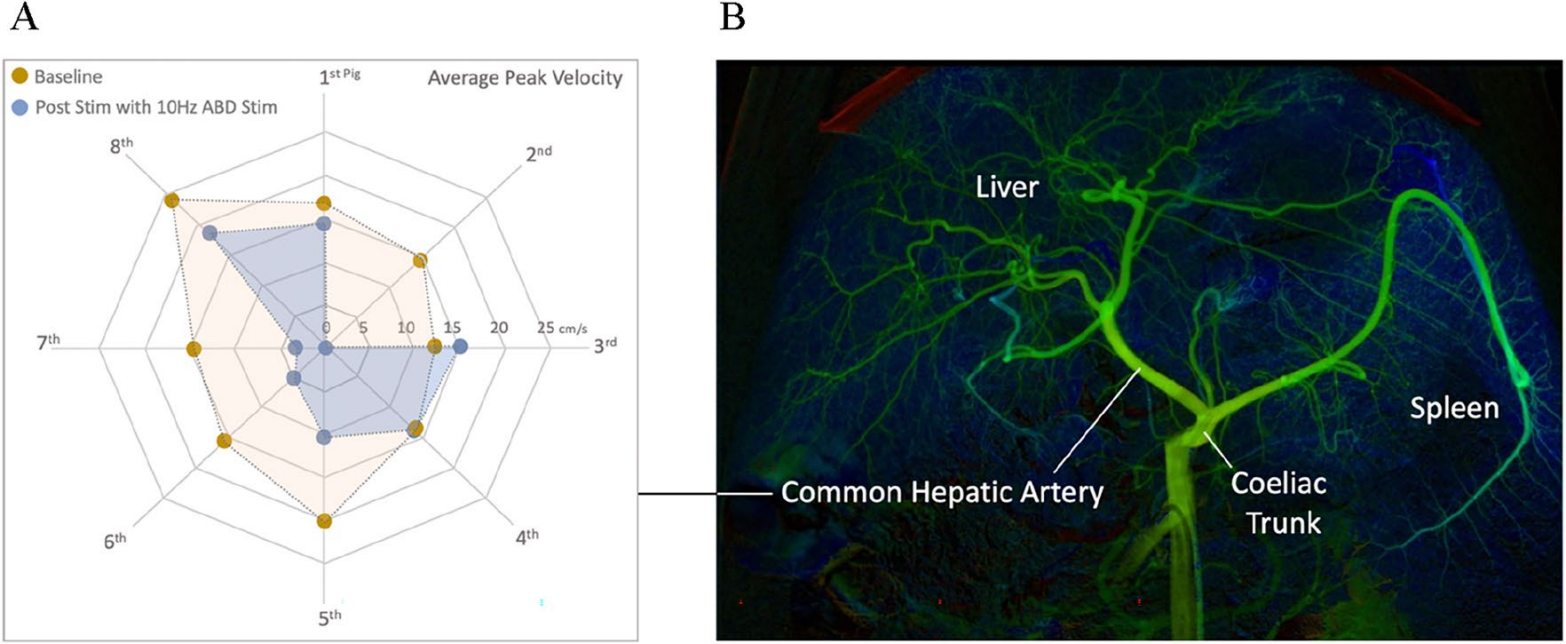
Common hepatic artery stimulation output. (**A**) Radar plot displaying the baseline and post stim response in APV to 10 Hz abdominal (ABD) stimulation (A10Hz) across the 8 pigs. (**B**) DSA image of the porcine arterial anatomy used as a guide for intra-arterial recordings of common hepatic artery.

**Figure 5:**
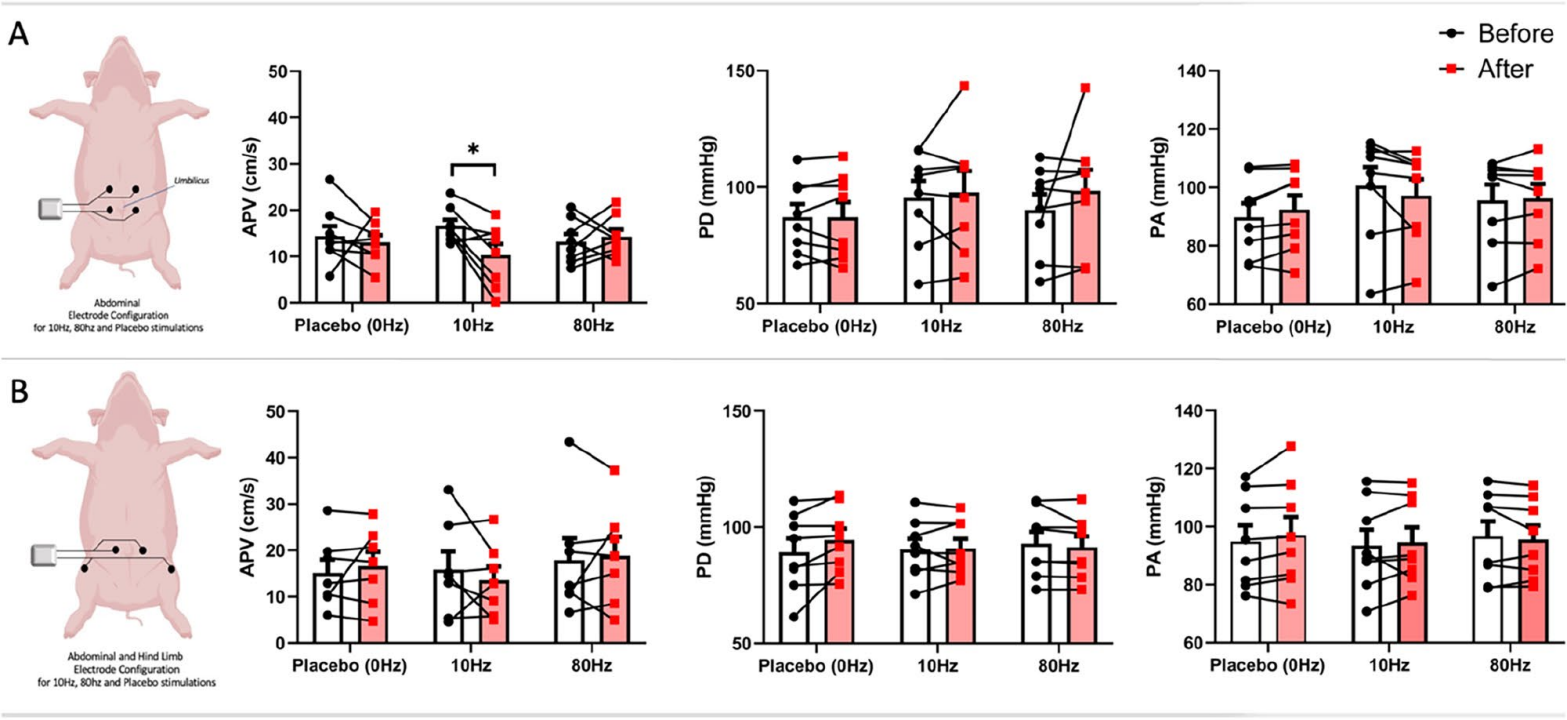
Common hepatic artery APV, PD and PA results with stimulation frequency and orientation. (**A**) Stimulation with the Abdomen-only configuration of TENS electrodes, (**B**) stimulation with the Abdomen-Hind limb configuration of TENS electrodes. *p < 0.05.

Of the six different stimulation conditions [abdominal placebo (AP), A10Hz, A80Hz, abdominal hind limb placebo (ALP), AL10Hz, AL80Hz] only the A10Hz stimulation demonstrated a statistically significant (p = 0.0233) reduction in average peak velocity (Figs. 4, 5) No significant changes in PD and PA in any of the other stimulation conditions were observed. Pooled results are provided in Fig. 5 for all the stimulation conditions and recorded parameters.

### Left renal artery

**Figure 6:**
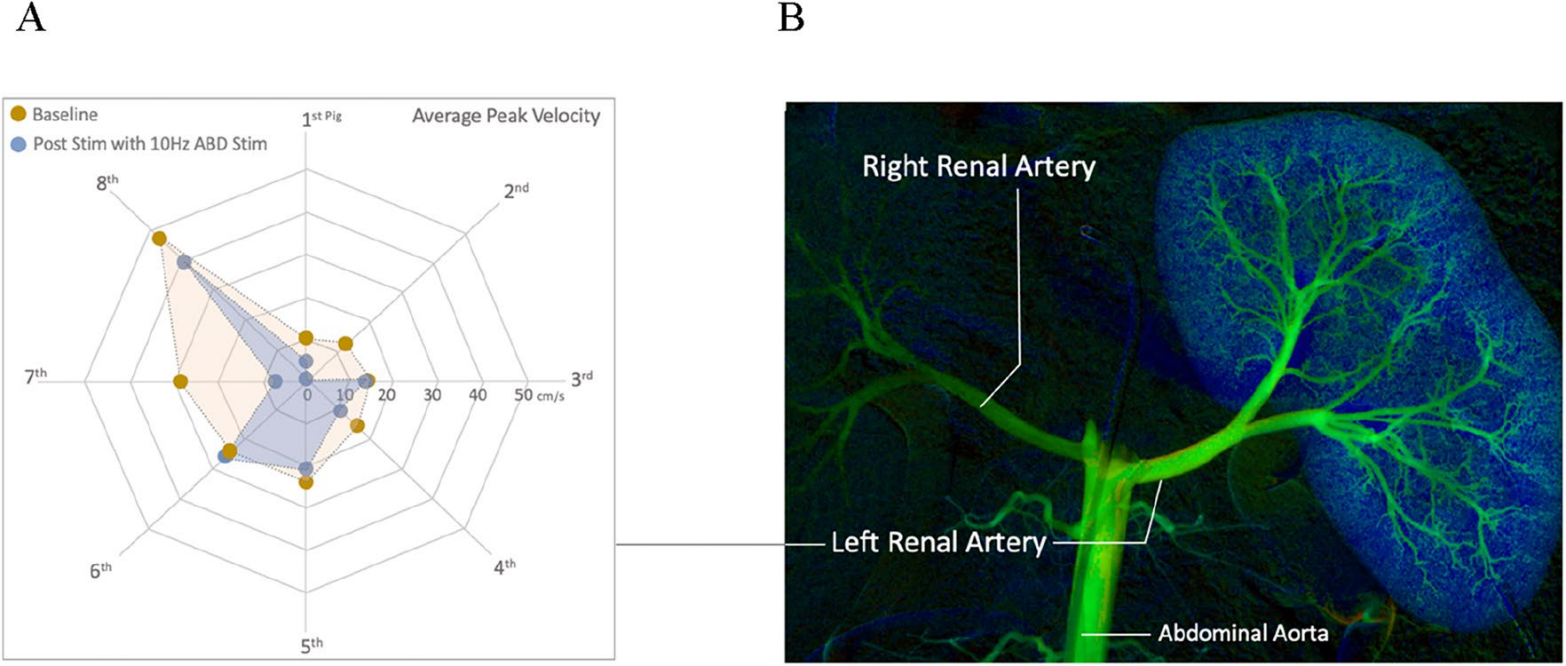
Renal artery stimulation output. (**A**) Radar plot displaying the baseline and post stim response in APV to 10Hz abdominal (ABD) stimulation (A10Hz) across the 8 pigs. (**B**) DSA image of the porcine arterial anatomy used as a guide for intra-arterial recordings of left renal artery.

**Figure 7:**
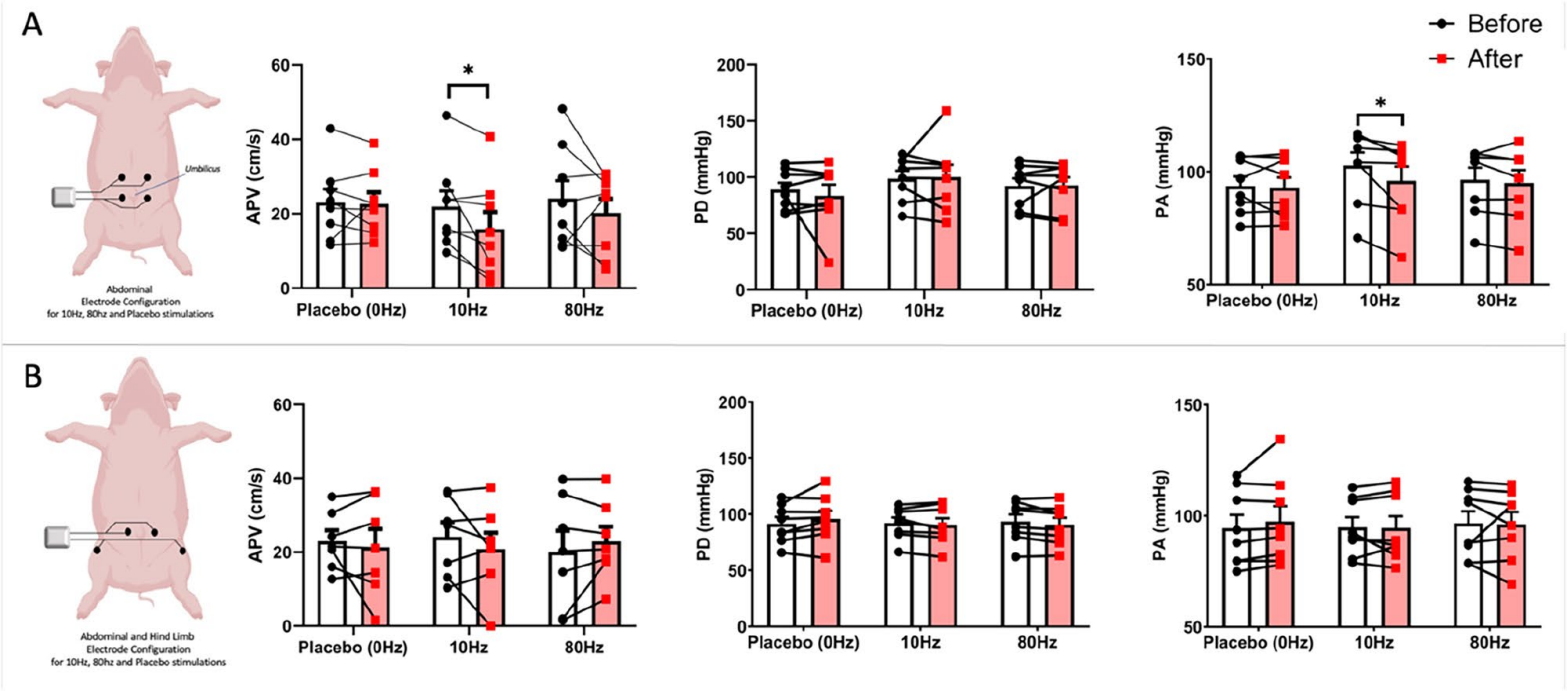
Common renal artery APV, PD and PA results with stimulation frequency and orientation. (**A**) Stimulation with the Abdomen-only configuration of TENS electrodes, (**B**) stimulation with the Abdomen-Hind limb configuration of TENS electrodes, *p < 0.05.

For the renal artery, the only statistically significance was observed for APV at A10Hz stimulation (p = 0.0493) (Figs. 6, 7). In addition, statistically significant (p = 0.0221) PA reduction was also observed with A10Hz. All stimulation conditions for renal artery and recordings for APV, PD and PA are shown in Fig. 7.

The six different stimulation conditions did not show any statistically significant change in localised pressure changes (PD) for any of the arteries (Figs. 5, 7, Supplementary Fig. 1). In addition, the systemic blood pressure changes with 10 Hz Abdominal stimulation was not observed in any of the common hepatic artery, anterior interventricular artery (Coronary) or extra/intracranial arterial recordings with any of the 6 different stimulation conditions (Figs. 5, 7, Supplementary Figs. 1, 2). The significance of this finding will be covered in the discussion session in the context of the renal artery ablation-based renal denervation to alleviate systemic hypertension. Left Coronary artery recordings from its anterior interventricular artery branch (investigated for a potential remote conditioning effect of the abdominal and abdominal-hindlimb stimulations) also did not show any statistically significant modulation of the APV, PD or PA (Supplementary Fig. 1).

### Extra cranial and intracranial cerebral arterial circulation

Extracranial and intracranial arterial blood flow recordings with contrast-based Syngo iflow peak times did not show any significant changes in any of the regions of interests with any of the 6 stimulation conditions. All of the peak time results are provided in Supplementary Fig. 2. In addition to peak time recordings for each extra and intracranial arteries, we also analysed arterial mean transit time from the ascending pharyngeal artery to the internal carotid artery for all 6 different stimulation conditions and none of the conditions demonstrated a statistically significant change in arterial mean transit time. The results of the arterial mean transit times are provided as a supplementary file (Supplementary Fig. 3).

## Discussion

The present study investigated the potential effects of non-invasive electrical stimulation with two different types of electrode configurations combined with the three different stimulation  conditions (2 different frequencies and a placebo) on supressing the non-compressible internal organs blood flow. Intraarterial blood flow recordings alongside intraarterial local and systemic blood pressure recordings were taken. To our knowledge, this is the first non-invasive large animal neurostimulation study that investigated multiple non-compressible internal organs’ blood flow modulation with intra-arterial catheter recordings.

Among 6 different stimulation combinations that have been investigated in the present study, the abdominal electrode configuration which is configured to correspond with segmental hepatic and renal innervation is the only stimulus protocol that has demonstrated statistically significant suppression of common hepatic artery’s and renal artery’s blood flow velocities (APV). In addition to specific effect of the electrode configuration, a frequency specific effect is also observed. 10 Hz Abdominal-only stimulation demonstrated the reduction of blood-flow of hepatic and renal arteries whereas 80 Hz stimulation with the same configuration of the electrodes could not achieve a statistically significant change in any of the parameters recorded including the blood flow velocity recordings. In the particular case of 10 Hz Abdominal-only stimulation, an almost total reduction of the post-stim blood flow velocity (APV) was also observed (Fig. 5A, AVP results). Although this reductions are found to be statistically significant, their potential clinical impacts should be investigated in internal bleeding models or blood-shift cases of humans to explore the clinically meaningful effects.

The statistically significant change in arterial pressure recordings corresponds with renal artery blood flow decrease in 10HZ stimulation in the context of renal denervation studies. Renal denervation to ablate the sympathetic innervation of the renal artery and kidney with adrenal gland has been trialled in multiple studies to investigate its potential therapeutic effect on intractable hypertension cases^22^. To date, the therapeutic outcome of these ablations’ studies are confounding and not consistent^23,24^. Furthermore, these ablation studies focused on long-term effects so their outcome and methodology are not comparable to the present study. However, it has been demonstrated that in normotensive, non-denervated anesthetized sheep, unilateral electric stimulation of the renal nerve reduced renal blood flow and it is also shown that electric stimulation^25^ of the renal nerve with lower frequencies (1 and 3 Hz) demonstrated less pronounced changes in renal blood flow (RBF) in comparison to a higher frequency (5 Hz) stimulation. Our present study also demonstrated the reduced RBF with 10 Hz non-invasive abdominal electrical stimulation which supports the higher frequency efficacy on the RBF, however, this modulation was not observed with 80 Hz abdominal stimulation which also indicates that increasing the frequency of stimulation to obtain a more pronounced effect may not be based on a linear relationship for the RBF modulation. In the same normotensive, non-denervated anesthetized sheep, unilateral electric stimulation of the whole renal nerve study, mean arterial pressure was also recorded. Although it was reported that the mean arterial pressure increased with unilateral electrical stimulation of renal nerve, such an increase was monitored only at the last 10 s of the 30 s of electrical stimulation and it was abolished in the following 20 s. In consideration to this fact, the blood pressure decrease that has been observed with reduced RBF in the present study was obtained with 10 mins long stimulations and post-stim recordings were also started 28 mins after the baseline recordings of renal artery (9 min after the stimulation, Fig. 2). In this context, continuous monitorisation of systemic blood pressure is essential to reveal the immediate, acute, and long-term effects on systemic blood pressure of non-invasive abdominal electrical stimulation.

The result of the present study indicates the specific combination of the stimulation frequency and electrode configuration is essential for the non-compressible internal organ blood flow suppression with non-invasive electrical stimulation. These results were also in line with our and other previous studies in humans that have demonstrated the specific effect of optimal dermatome and frequency combination on internal organ blood flow modulation^1–5,7^. In a previous study, our group demonstrated that the 10 Hz was capable of modulating the testicular artery blood flow with the electrodes configured on the T12 dermatome in humans^7^. However, the same stimulation frequency was incapable of modulating the blood flow of the testicular artery when the electrodes were configured on the T10 dermatome^7^.

### Potential mechanism of actions

In previous studies by our group and others, dermatomal stimulation configured corresponding to the segmental sympathetic innervation of the visceral organ arteries was found to be an effective design to modulate the targeted internal organ’s blood flow including ovaries, uterus, and testicles in humans and animal studies^1–5,7^. The present study stimulation frequencies (10 Hz and 80 Hz) were chosen in the context of previous blood-flow neuromodulation studies^1–5,7^. In addition, the abdominal-only configuration is also designed to correspond with hepatic and renal sympathetic innervation as explained with the detailed anatomy in the methodology section. The present study results were in line with the specific segmental electrode configuration (Abdominal-only) is needed to interact with corresponding internal organs when non-invasive neuromodulation is utilised. However, other potential mechanisms cannot be ruled out. In addition to segmental mechanisms, a supraspinal mechanism has also been postulated as one of the mechanism of action for non-invasive internal organ’s blood flow modulation^1–3^. Angiosome innervation can also be considered for the neural pathway as an additional mechanism of action. Angiosomes are the skin's arterial territories. In this context, a potential mechanism of action via innervation of angiosome can also be considered for abdomen only electrode configuration with 10 Hz stimulation efficacy on the common hepatic artery blood flow suppression. The abdominal-only configuration comprise the upper abdominal skin and upper abdominal skin arteries’ (angiosome of internal thoracic artery and its superior epigastric artery branch) sympathetic innervation originates from the stellate, cervical sympathetic and upper thoracic(T1–T2) ganglions^26^. Sympathetic nerves that originate from the stellate ganglion and cervical ganglions hijack the vagus nerve and descends within the vagus nerve to the abdomen and leaves the vagus to innervate the abdominal ganglia^27^. It has also been demonstrated that abdominal ganglions innervate the corresponding vasculature^28^. As such abdominal-only stimulation with its upper abdomen electrode configuration (not the lower abdomen and leg configuration) has the potential to stimulate upper abdominal angiosome via sympathetic innervation. This in turn can modulate the stellate/cervical ganglion where the modulation of stellate/cervical ganglion has the potential to relay to abdominal ganglia and modulate abdominal arteries’ blood flow. Finally, it has been demonstrated that moderately high intensity of direct arterial electrical stimulation is capable of suppressing the blood flow and contracting the arteries^10–12^. With this in mind, a fourth potential mechanism of a direct electrical field effect of non-invasive electrical stimulation on abdominal arteries can be not excluded in the context of the high intensity (35 mA) that has been utilised in the present study as well as the previous study results used invasive arterial electrostimulation at high intensity and 10Hz for vasoconstriction^11^. Future denervation studies are essential to demonstrate the exact mechanism(s) of action.

Although the present study incorporated some cutting-edge technologies of blood flow monitorisation of multiple internal organs in vivo and intra-arterial recordings as well as the baseline and post-stim monitorisation consistency of the methodology (keeping the tip of the catheters in the same place by using the baseline recordings X-ray images), it also has limitations and also opens new questions for further research. The experiments and monitorisation of blood flow were acute and long-term effects (including potential rebound effects) of non-invasive neuromodulation on internal organ blood flow should be investigated with further studies. Internal organs’ blood flow is monitored unilaterally for kidney, coronary arteries and carotid arteries, and hence bilateral responses should be monitored with further investigations. 35 mA intensity is used in the present protocol but the effects of different intensities in the same protocol need to be investigated as well. Because of the sensor localisation of the flow catheters, short arterial trunks like coeliac trunk could not be investigated so whether the common hepatic arterial blood flow modulation can be extrapolated to spleen and gastric blood flow is still an unknown paradigm. The blood-flow changes in the renal artery were lower than the blood-flow changes that are observed in liver in abdomen-only configuration. This may be due multiple factors. First, the anatomical segmental sympathetic innervation differences in these organs and the segments that are stimulated by the abdomen-only configuration comprise more segments (as stated in the methodology) for liver innervation but less for kidney. The second factor could be due to the intrinsic blood flow differences in between these organs. The liver has dual-arterial inflow (hepatic artery and portal vein) whereas kidney has a single arterial inflow (renal artery). We also acknowledge that the changes observed in the present study cannot be directly extrapolated to human or clinical applications. The present study only demonstrates the potential of TENS in the blood flow modulation of these organs and future studies are needed on whether the prolonged-term applications are feasible to be able to maintain long-term blood flow reduction in humans that are clinically meaningful. Furthermore, the blood-flow measurements were taken pre and post stimulation to remove the variability of *stimulation duration* for each organ. We utilised a 10-min stimulation window to stabilise stimulation periods. On the other hand, the period in between the pre and post stimulation recordings for each organ were different because of the time required for catheter to move in between different organ arteries. In this context, a potential earlier or later response of the blood flow changes of each organ cannot be ruled out. In addition, the pressure changes reported in the present study may also occur at varying times compared to the flow changes. The venous blood responses are also another lack of the present protocol but it is also a significant aspect that should be the focus of the following studies. We incorporated an anaesthesiologic method that was previously demonstrated to have negligible effects on vascular tone and blood pressure, however, the potentially different responses of the vascular system in an awake organism cannot be excluded. It is worth noting that this small-scale exploratory study did not account for p-value adjustments for multiple comparisons. While this was the case the Fisher's LSD tests provided the necessary power to show differences in our study, it is reported that Fisher LSD preserves the type I error rate at the nominal level of significance if only the number of treatment groups is three^29^ (10Hz, 80Hz, and Placebo for the present study). It is also indicated that multiple comparison adjustments are not necessary for exploratory studies but for confirmatory studies^30,31^. Finally, while p-value adjustments decrease the chance of making type I errors, they also increase the chance of making type II errors or needing to increase the sample size^30^. In the future, a larger sample size will be considered for a large-scale confirmatory study.

## Conclusion

Overall, the present study provided first insights of frequency and dermatome specific effects of abdominal non-invasive transcutaneous electrical stimulation on non-compressible organs of liver and kidney blood flow. The results also demonstrated the transcutaneous abdominal electrical stimulation is capable of reducing the hepatic and renal blood flow (as well as PA) with dermatome and frequency-specific effects. The reductions in blood flow seen in this study highlight the capability of TENS stimulation to be utilised in potential clinical scenarios including non-compressible internal organ bleedings to provide additional time for medical help to reach remote regions of injury. It is also important to consider that the present study is performed in pigs and whether the blood flow reductions observed are sufficient to be clinically meaningful or not, still needs to be investigated in human clinical conditions.

### Supplementary Information

Supplementary Information.

## Data Availability

The datasets generated during the current study are not publicly available or for secondary analyses due to the restrictions of the ethical approval. Data are however available from the corresponding author upon reasonable request and with permission of ethical committee.
