# Supplementary material for: Decreasing the blood flow of non-compressible intra-abdominal organs with non-invasive transcutaneous electrical stimulation
Source: Sci Rep. 2024 May 2;14:10122. doi: 10.1038/s41598-024-55165-8 (PMC11066058; doi:10.1038/s41598-024-55165-8)
Supplement: Supplementary file 1 — Supplementary Information. [file 41598_2024_55165_MOESM1_ESM.pdf]

Supplementary files

Methods

Experimental Protocol

During the sedation and experimental protocol, a number of parameters were monitored to ensure that they were within the normal ranges. Hartmann's solution was infused intravenously at 5 ml/kg throughout anaesthesia to maintain adequate hydration status. Vital signs (pulse oximetry, invasive blood pressure, heart rate, electrocardiogram, end tidal carbon dioxide, and oesophageal temperature) were continuously monitored throughout the procedure (IntelliVue Mx800 system, Philips Healthcare, Australia). Following securement of vascular access, heparin was administered at 100 – 300 iu/kg intravenously as required to maintain an activated clotting time of greater than 250 seconds for the remainder of the procedure.

Intracranial and extracranial blood flow Analysis with DSA-Syngo iflow

In pigs, the rete mirabile caroticum (paper Figure 3) blocks the arterial passage at the distal end of the ascending pharyngeal artery so the catheters cannot reach the internal carotid artery. Hence, flow catheters are not an option to monitor blood flow changes in the internal carotid artery and that is why iflow contrast techniques were utilized.

Coronary Artery & Carotid Artery Results

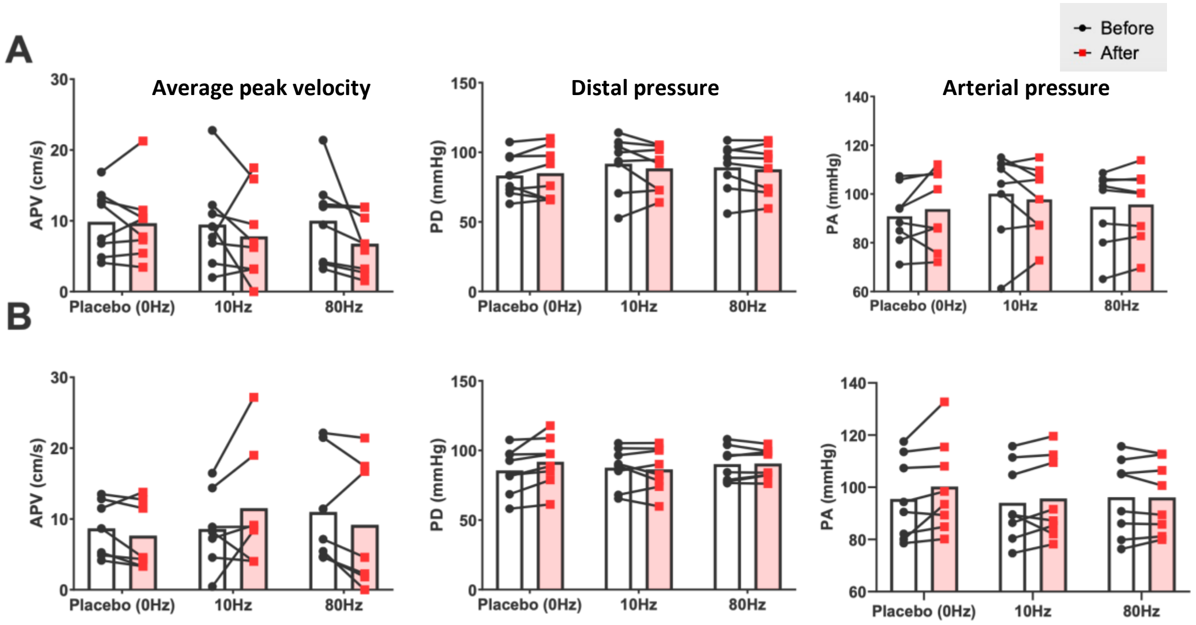

Supplementary Figure 1: LAD-Anterior Interventricular Artery (Left Coronary Artery) APV, PD and PA results with stimulation frequency and orientation A: Stimulation with the Abdomen-only configuration of TENS electrodes, B = Stimulation with the Abdomne-HindLimb configuration of TENS electrodes

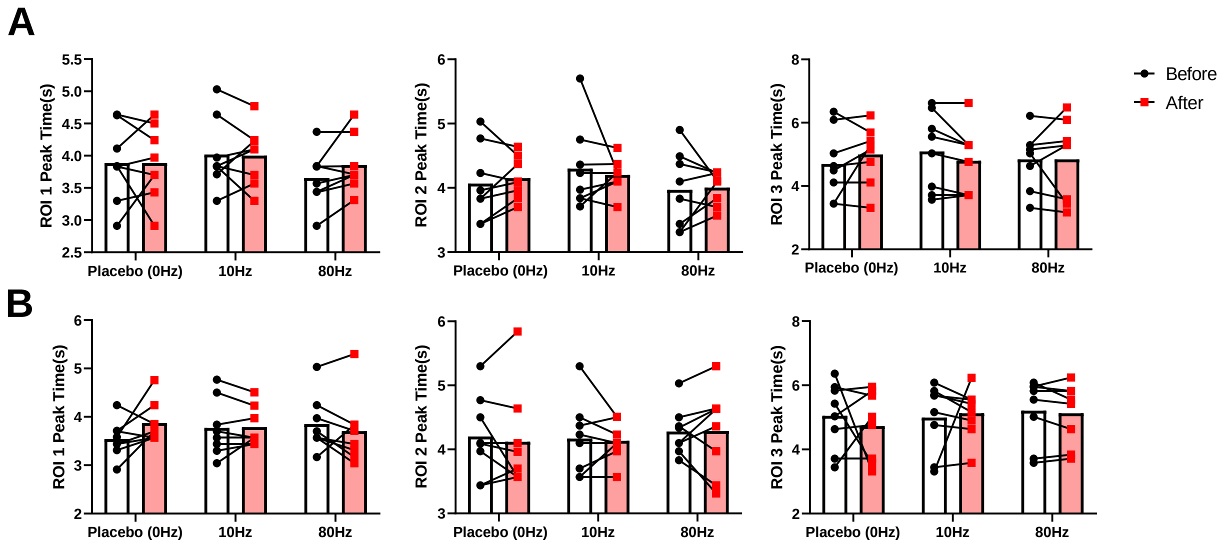

Supplementary Figure 2: Carotid artery ROI peak times taken from iFlow A: Stimulation with the Abdomen-only configuration of TENS electrodes, B = Stimulation with the Abdomen-HindLimb configuration of TENS electrodes. ROI 1-3 are defined and demonstrated in the Figure 3 of the main manuscript.

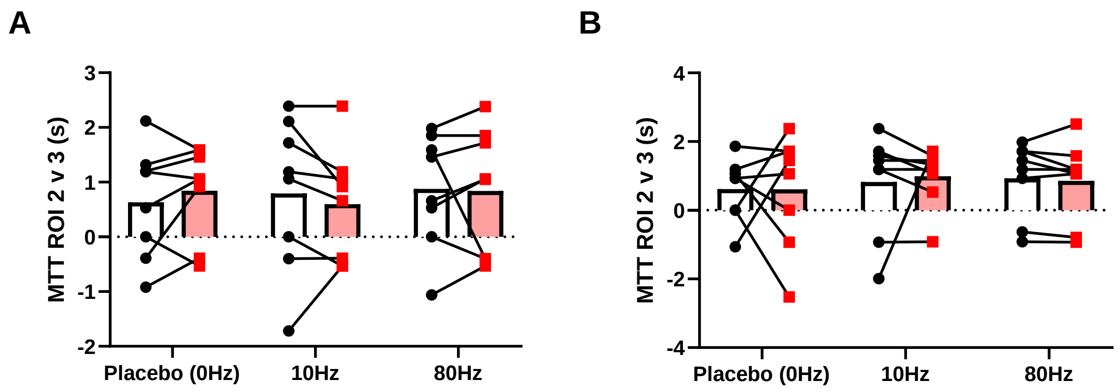

Supplementary Figure 3: Syngo iflow ROI2 and ROI3 based (Cerebral Arterial Circulation) Mean Transit Time(MTT). MTT is measured as the time difference between each of the ROI2 and ROI3 peaks times i.e. ROI3 minus ROI2 time. In a few of the pigs, the ROI3 peak time was faster than ROI2 and this may be due to multiple factors including anatomical collateral vessel variations and the rete mirabile. A: Stimulation with the Abdomen-only configuration of TENS electrodes, B = Stimulation with the Abdomen-Hindlimb configuration of TENS electrodes. ROI 2-3 are defined and demonstrated in the Figure 3 of the main manuscript.
